# Notes from Dispensary and Private Practice

**Published:** 1877-10

**Authors:** 


### NOTES FROM DISPENSARY AND PRIVATE PRACTICE.

*Rare Form of Congenital, Multiple amd Monolateral Pigmentary Nœvus, having the disposition of Zoster Corporis.*

On the 18th of August, 1877, John McDonald, an unmarried laboring man, aged 31 years, native of St. Johns, New Brunswick, applied at the skin department of the South Side Dispensary of Chicago. His stature was rather above the medium height, hair and eyes black, complexion florid. He stated that his father and mother were healthy, and that he had twelve brothers and sisters none of whom exhibited congenital deformity.

Since his arrival at adult years he had always indulged freely in the use of spirits until the beginning of the present year. In 1867 he contracted a gonorrhœa which lasted for three months, and was accompanied by an attack pronounced by his physician to be "gonorrhœal rheumatism." One year afterward a suspicious intercourse was followed by swellings in each groin, unaccompanied by any observed genital lesion, which suppurated and were incised. In three months thereafter, intercourse was again succeeded by suppurative inguinal adenopathy, and an ulcer upon the glans penis. On the third year afterward he again had genital ulcer and non-suppurative swellings in the groin. In 1875, when suffering from an eruption produced by the poison ivy, he was treated by Dr. F. W. Draper, in the Boston City Hospital, and relieved, as he states, by the application of zinc ointment. Soon after he was treated for blotches upon the face, which were followed by pustules, pronounced by Dr. Chandler to be lesions of secondary syphilis.

In the latter part of July, 1877, after laboring in the grain fields of Sterling, Illinois, he suddenly noticed that his face and hands were "burning hot," and soon became much swollen, little "watery heads" appearing over the affected parts. This led him to apply for relief.

On examination, the exposed surfaces of the body were found to be affected with what seemed to be an induced eczema in the phase of retrogression. The skin of the face was erythematous merely—the swelling described having subsided, while the outer aspect of the upper extremities, and the wrists in particular, were reddened, excoriated and covered with light yellow crusts. The portions of the integument covered by clothing were entirely unaffected, except as mentioned below: the skin being soft and white, and exhibiting no cicatrices except those resulting from the old inguinal incisions. The hair was abundant; there was no adenopathy; the fauces were unaffected, and no trace of syphilitic disease was discernible. It seemed probable that the patient had again suffered from the poisonous effects of the rhus toxicodendron, and such in fact was his own belief. In a few days the cutaneous disease disappeared under the use of an emollient lotion.

The examination of his person led to the incidental discovery of a unique deformity which he stated had existed from birth, and which, his mother had told him, she had for a long time after he was born, endeavored to remove by washing, on the supposition that his skin was covered with "dirt." Its appearance had not changed since he first began to notice it, and it was unaccompanied by subjective sensations.

The deformity existed exclusively upon the left side of the body, and was produced by the abundant development of uniformly split-pea sized, irregularly square-shaped, discrete and closely packed, pigmentary nævi, varying in color from a light fawn to a deep chocolate, with the exception of the scrotal region, where the moles were larger and of a dull reddish hue.

The flattened summit of each was slightly uneven, elevated above the surface of the integument by about one-eighth of an inch, and in no single instance provided with hair.

Four distinct regions of distribution could be defined as follows :

1. A single line of closely-packed moles, commencing in the axilla, at about one-half inch posterior to the axillary border of the pectoralis major muscle, extended in a gentle curve, through a point one-half inch above the left nipple, to the exact mesian line of the sternum, where it abruptly terminated. These were of a light fawn color, and were the lightest in shade of all displayed upon the person.

2. Over the 8th rib, and beginning at a line dropped from the anterior border of the axilla, a broad ribbon of deep chocolate-colored, and in some places almost black, nævi, densely crowded together, extended in a gentle curve exactly to the linea alba, spreading thence upward and downward, in a vertical line, from the umbilicus to six inches above that point. This T shaped band was, on an average, one and one-half inches in width.

3. Commencing over the latero-posterior region of the trunk, five inches from the lumbar spines and one and one-half inches above the dorsum of the ilium, another ribbon, composed of closely-packed moles, extended horizontally to an imaginary line dropped from the anterior border of the axilla. This was the sole region where the hypertrophic growths did not fully extend to the median line of the body. They were of a deep fawn color, lighter in shade than these last described.

4. Beginning at a point about midway between the anus and the left tuber ischii, an irregular line of nævi extended forward and upward over the left side of the penis and scrotum, where they were disposed without apparent order. These were, as has been stated, larger than those upon the trunk, being many of them of the size of a small bean, and displaying a dull red hue. In no instance was one discovered on the right side of the raphé of the scrotum, nor upon the right of the mesian line of the penis. The nævi upon the penis were smaller and much less pigmented than those upon the scrotum.

All the extremities were free from the deformity.

Viewing the patient at a distance sufficient to remove the distinction of individual nævi, and observing merely the lines and areas traced by the hypertrophy, the appearance was highly suggestive of zoster of the trunk. The patient stated, moreover, that his former physicians had declared that he was affected with "shingles."

Excision of a portion of the hypertrophic growths for further examination was not permitted.

According to Hebra, the existence of pigmentary moles is rarely established at birth. The history of the case here reported is quite conclusive as to the congenital character of the lesions, and their distribution indicates, very distinctly, that the affected surfaces corresponded to the regions of skin supplied by the anterior branches of the lateral cutaneous nerves. The fact that certain maternal nævi are due to intra-uterine perturbations of the nervous ganglia of the fœtus, was first observed by von Baerensprung in 1859(1), and embodied in a paper by the same author in 1863. An interesting communication by Dr. Roberto Campana of Naples, to the *Italian Journal of Venereal and Skin Diseases* for October, 1876(2), establishes, further, an intimate connection between the location of *vascular nævi* and the territories supplied by various cranial, cervical, brachial, dorsal and lumbar nerves. The fact of such intra-uterine involvement of the nervous ganglia of the fœtus will at present be scarcely disputed; but I have been unable to find a reported case which, in its general features, resembles that described above. It, however, most nearly corresponds to an observation made by Martin Arndt in 1839. In the report made by the latter, it appears that the left side of the body was exclusively affected, pigmented, hypertrophic nævi, isolated and occurring also in bands, having been observed upon the neck, the shoulders, the clavicle, the upper arm, the elbow and the thorax. In the locality last named, three parallel bands of deeply pigmented nævi extended to the median line of the body, and there

---

(1) Nævius unius lateris Von Baerensprung, Ann. d. Char. Krank., Bd 11, Sept. 2, 1863, p. 91, et seq.

(2) Giorn. Ital. d. Mal. Ven. e. d. Pel., Oct., 1876; F. 5, p. 257.

coalescing, spread in a vertical direction to the umbilical cicatrix.

The latest reported instance of such hypertrophy was observed by De Amicis, in 1876[1], upon the person of a dark brunette, aged 17 years. But here the entire cutaneous surface was covered with disseminated blackish-brown pigmented nævi, the extremities, including palms and soles, being invaded. The deformity was not described as congenital.

JAMES NEVINS HYDE.

CHICAGO, August, 1877.

---

*A Case of Puncture of the Intestines for the Relief of Over-distension with Gas.*

In the JOURNAL AND EXAMINER, Oct., 1876, I reported a case in which I had punctured the bowel for the relief of enormous distension, with marked beneficial results.

I will now give a short history of another case where the same treatment was adopted, and though there was ultimately an unsuccessful issue, it in no wise reflected on the beneficial results of this treatment.

I was called, on the 6th of July, to see D. A., aged fifteen years. He had been playing ball a couple of days before, and, while heated from this exercise, drank freely of cold water.

On examination, I found his pulse 108, tongue thickly coated, creamy-yellow, tenderness over the whole abdomen, but more particularly in the region of the sigmoid flexure of the colon, persistent vomiting and considerable pain. I diagnosed the case as one of colitis, and ordered some powders of morphine and bismuth, warm poultices to be applied over the abdomen. I visited the boy daily, and although the pulse rose to 120 with tympanites and a more extended area of tenderness by the 13th, there was a general amelioration of the morbid symptoms, and I regarded him as convalescent. On

(1) Lo Sperimentale, Mar., 1876, p. 312 (Archives of Dermatology, III, II, p. 158.)

the evening of 14th I was sent for in haste to see him. The messenger said he was "taken with cramps in his bowels," and was suffering great pain. Being otherwise engaged, I did not see him that night; but ordered ¼ gr. morphia every two hours to relieve pain, and poultices to be re-applied. Next morning, on visiting him, I found his pulse had risen to 120, and was feeble; tympanites had increased, so that the distension was oppressive; pinched expression of countenance—in short, all the symptoms indicating general peritonitis, which, in fact, was the case. I ordered the morphine and poultices to be continued; but he gradually grew worse, so that by the morning of the 17th his pulse had risen to 140, and was quite feeble. The distension was so great that he had great difficulty in breathing from the pressure on his lungs; the pupils were dilated, skin beginning to get cold, and loss of nervous sensibility, indicating approaching collapse. I had previously determined that, all other means failing, I should resort to puncture and directly liberate the pent-up gas, and I thought the time had now come when it would be proper to do so. Accordingly, I inserted a hollow needle in the region of the transverse colon, liberating a quantity of very offensive gas. I again inserted the needle in the epigastric region, with the effect of permitting the escape of a much greater quantity of gas than from the first puncture, and very sensibly diminishing the distension. *His pulse fell eighteen beats per minute before I left the house*, and the distension never afterwards rose to the same height. Next day his pulse was 118, and tympanites considerably reduced. He continued to improve for several days, when he had some diarrhœa and fever; bnt these symptoms were successfully combatted without much difficulty. He now progressed slowly towards recovery, so that by the 3d August he was able to sit up. By this time his tongue had become clean, and the pulse had fallen to about ninety, which, considering his emaciated condition, was fair. His appetite now became ravenous, and I duly cautioned his attendants not to permit him to eat much at a time, and prescribed the food which I considered best suited to his condition. Notwithstanding my care in this particular, on the evening of 9th August, he was permitted to eat heartily of "hash," composed of potatoes, onions, beef, etc. Some four or five hours after partaking of this combination, he was seized with violent pain in the bowels, accompanied by vomiting; and in his debilitated condition, he made but little resistance, so that by the next day evening his condition was hopeless, and he died the same night.

I publish this case because I think it is of the utmost importance that this treatment of puncturing the bowel in case of distension, should have a more extended trial. Heretofore our efforts have been limited to fomentations and internal remedies, and these failing, as they so often do, there is no reason why it—i. e. puncture—should not be tried, especially as it has been demonstrated, over and over again, that it is not such a dangerous procedure to puncture the peritoneum. This boy would undoubtedly have died on July 17th, but for the measures which were taken.

E. W. Lee, M. D.

417 W. Jackson St., September, 1877.

---

*Electricity in the treatment of opium poisoning.*

At 7 o'clock a. m., March 27th, I was called to see Mrs. S., aged sixty-five years, who was suffering from excruciating spasmodic pain in her stomach, accompanied by frequent belching of gas. She had been subject to such attacks and accustomed to the use of opium for their relief, though she had not found it necessary to take any for more than a year. She had at hand a solution of the deodorized tincture of opium, as well as paragoric, so I administered 60 drops of the former and two drachms of the latter, with a drachm of the bicarbonite of sodium, after which I returned to my office. I visited the lady again at 8.30 and found her still suffering and almost delirious from pain, anxious to find relief in death. I prescribed a drachm of ipecacuanha as an emetic, which failed to vomit, and in fifteen minutes she was asleep. I was called into the country, aud did not return until 2 p. m., when I was hastily summoned to see her, because she had not been awake since 9 a. m., and could not be aroused. If there is ever a time when a physician realizes his responsibility it is when a patient is dangerously narcotized and he has ordered the medicine which may have produced it. I attempted to administer strong coffee and stimulants, of which she could swallow but little, and within an hour all efforts in this direction were useless, as no manipulation would induce her to swallow. Her respirations at this time were six per minute; pulse, fifty and weak; pupils contracted; skin relaxed, with a clammy perspiration and stertorous breathing. In my dilemma, I resolved to try electricity. I applied the battery at 4 o'clock, and repeated it every 15 or 20 minutes, using it for about five minutes at a time, directing the current from the back of the neck and along the spinal column to the feet, and also to the breast, stomach and bowels; as near as possible along the course of the pneumogastric nerve. The attendants frequently rubbed the surface of the body and limbs with strong mustard and water. The effect of the battery was the drying up of the perspiration and a warm feeling of the skin. The throat was frequently cleared of mucus by a swab. After eight hours persistent treatment she moved one of her hands, and in an hour could be induced to swallow, and during the next two hours we gave a pint of strong coffee and half a pint of gin. She made a good recovery, and in a week was able to be up again, and is now engaged in her usual avocations.

Physicians are sometimes undeservedly censured, and in this case, had Mrs S. died, I should have been blamed for causing her death. The facts in the case, however, are that Mrs. S. had taken, at 3 o'clock in the morning, an ounce of the preparation prescribed; that is, half an ounce of the opium solution and half an ounce of paragoric, a fact of which I was then ignorant. Owing to the congested condition of the stomach, the medicine was not absorbed until I gave the ipecac, which relaxed the organs and the opium soon produced a dangerous narcotism.

WYONET, Ill. F. C. ROBINSON, M. D.

